# In an Urban District Infectious Hospital

**Published:** 1914-05-09

**Authors:** 


### The Patients' Column.

### IN AN URBAN DISTRICT INFECTIOUS HOSPITAL.

A very pleasant nurse came in the ambulance to fetch me, and I was wrapped in a blanket and driven to the Urban District Council's Hospital. Mine was a light case of scarlet fever. I was not feeling very ill and walked from the ambulance to the ward, where I was laid outside the bed with a screen round me. Sister arrived and looked at me, and I was then given a blanket bath. When the screen was removed I had time to look round the ward, which had eight beds in it, the other seven being occupied by children. I was later visited by the doctor and the matron and duly certified. Lights for the children were out at 6.30, but I was allowed a screen and a light to read by until later. At about nine I slept a little, but was awakened about eleven by the night nurse, who was vigorously cleaning the lockers, moving them noisily for the purpose. After she had finished she addressed me thus: "Now, Mrs. What's-your-name, where are you? Oh, there you are! Got all your clothes to mark; run off my feet as it is." This nurse fortunately left the hospital a week or two later. She used to make the children cry at night by refusing to let them have the bed-pan when they asked for it, and generally spoke to them very angrily, "Don't suppose I am here to attend you at all hours of the night." She addressed me first as if I had been a charwoman, but later became very confidential, and told me about herself and her friends. I was awakened at 1.30 the first morning to be washed for the day. The children were often washed at 2 A.M., and were most of them asleep during the process.

Happily the nurses were not all like this, and, generally speaking, were kind to the patients. I noticed that the social status of the nursing staff all round was lower than one meets in a general hospital. The training of most of them seemed to be in its early stage, and hardly any of them could do things for the patients gently or nicely. Their chief aim appeared to be to make a bed in hospital fashion. Although strictly forbidden to do so, the nurses often used to kiss and fondle the children.

The sister had a considerable sense of her position, and rebuked us if we did not stand up when she entered the ward or passed us in the grounds. She unbent, however, occasionally, and liked a good gossip. She was rather addicted to running down her fellow-workers.

It was not long before I was allowed to go into the grounds for the greater part of the day. We were all warned of the danger of going near the diphtheria block. A little boy said one day when refused something, "If you don't give it me I shall go and catch dip."

To each bed was a set of utensils, etc., for the sole use of the patient in the bed. But the washing bowls in which the patients soaked their feet were often taken by the ward-maid to wash up the tea-things in.

The food was good and plentiful, but rather "thrown at" one. Some nurses could make a good cup of tea, but others failed in this important matter.

